# Analysis of Antibody Neutralisation Activity against SARS-CoV-2 Variants and Seasonal Human Coronaviruses NL63, HKU1, and 229E Induced by Three Different COVID-19 Vaccine Platforms

**DOI:** 10.3390/vaccines11010058

**Published:** 2022-12-27

**Authors:** Diego Cantoni, Gabriel Siracusano, Martin Mayora-Neto, Claudia Pastori, Tobia Fantoni, Spyros Lytras, Cecilia Di Genova, Joseph Hughes, on behalf of the Ambulatorio Medico San Luca Villanuova Group, Lucia Lopalco, Nigel Temperton

**Affiliations:** 1 Viral Pseudotype Unit, Medway School of Pharmacy, The Universities of Kent and Greenwich at Medway, Chatham ME4 4TB, UK; 2 Division of Immunology, Transplantation and Infectious Disease, Immunobiology of HIV Group, San Raffaele Scientific Institute, 20132 Milan, Italy; 3 Department of Neurosciences, Biomedicine and Movement Sciences, University of Verona, 37129 Verona, Italy; 4 MRC-Centre for Virus Research, University of Glasgow, Glasgow G12 BQQ, UK; 5 Ambulatorio Medico San Luca Villanuova, Villanuova sul Clisi, 25089 Brescia, Italy

**Keywords:** SARS-CoV-2, seasonal, HKU1, 229E, NL63, neutralisation

## Abstract

Coronaviruses infections, culminating in the recent severe acute respiratory syndrome coronavirus 2 (SARS-CoV-2) pandemic beginning in 2019, have highlighted the importance of effective vaccines to induce an antibody response with cross-neutralizing activity. COVID-19 vaccines have been rapidly developed to reduce the burden of SARS-CoV-2 infections and disease severity. Cross-protection from seasonal human coronaviruses (hCoVs) infections has been hypothesized but is still controversial. Here, we investigated the neutralizing activity against ancestral SARS-CoV-2 and the variants of concern (VOCs) in individuals vaccinated with two doses of either BNT162b2, mRNA-1273, or AZD1222, with or without a history of SARS-CoV-2 infection. Antibody neutralizing activity to SARS-CoV-2 and the VOCs was higher in BNT162b2-vaccinated subjects who were previously infected with SARS-CoV-2 and conferred broad-spectrum protection. The Omicron BA.1 variant was the most resistant among the VOCs. COVID-19 vaccination did not confer protection against hCoV-HKU1. Conversely, antibodies induced by mRNA-1273 vaccination displayed a boosting in their neutralizing activity against hCoV-NL63, whereas AZD1222 vaccination increased antibody neutralization against hCoV-229E, suggesting potential differences in antigenicity and immunogenicity of the different spike constructs used between various vaccination platforms. These data would suggest that there may be shared epitopes between the HCoVs and SARS-CoV-2 spike proteins.

## 1. Introduction

In December 2019, the outbreak of a novel coronavirus named severe acute respiratory syndrome coronavirus 2 (SARS-CoV-2) rapidly spread around the world, resulting in a global pandemic [1]. Since then, international efforts to generate a suitable therapeutic have resulted in the development of multiple vaccination platforms and other antiviral pharmaceuticals. The gradual rise of variants has had a reduced impact on the efficacy of neutralising antibodies raised either by previous infection of SARS-CoV-2 or by vaccination [2,3]. The World Health Organisation (WHO) has categorised the troubling variants as variants of concern (VOC), whereas other variants that do not meet the same criteria fall under variants of interest (VOI) or variants under investigation (VUI). There has been a substantial amount of focus on variants and their characteristics, such as antibody evasion and replication rates, with many studies comparing variants and their ability to be neutralised [4,5,6,7,8], as the pandemic continues to progress.

**Figure 1:**
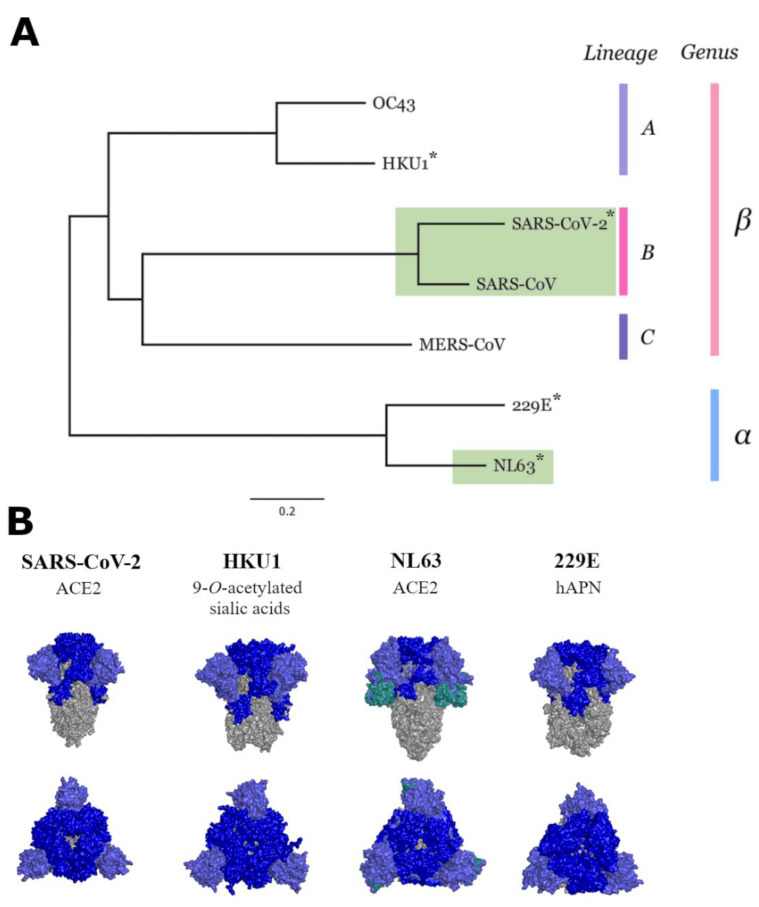
Phylogenetic tree of the members in the *Coronaviridae* family. * denotes spike proteins that were used in this study (**A**). Structures of spike proteins of SARS-CoV-2, and three of the seasonal HCoVs; HKU1, NL63 and 229E, which were used in this study (**B**). (PDB codes: 6VXX, 60HW, 6U7H, 5I08, and 5SZS). Grey denotes the S2 domain, whereas light blue is the N-terminal domain of S1 subunit, and dark blue represents the remaining S1 subunit. NL63 has an additional teal coloured section representing a unique region in the S1 domain not observed in other coronaviruses [21].

SARS-CoV-2 belongs to the *Coronaviridae* family that includes SARS-CoV-1 [9], middle eastern respiratory virus (MERS) [10] and four human coronaviruses 229E, HKU-1, NL63, and OC43 [11] (Figure 1A). Whilst SARS-CoV-1 and MERS have had outbreaks that caused severe disease in humans [12], the four other coronaviruses, commonly referred to as seasonal or human coronaviruses (HCoVs), typically cause mild disease similar to a common cold [11,13]. On rare occasions, however, the HCoVs may cause severe diseases [14,15,16]. SARS-CoV-2, together with NL63, use angiotensin-converting enzyme 2 (ACE2) as their major cell entry receptor [17,18]. Despite HKU1 and OC43 being more closely related to SARS-CoV-2, they bind to sialic acids as a mode of entry [19], whereas more distantly related 229E uses human aminopeptidase (hAPN) [20]. (Figure 1B).

**Figure 2:**
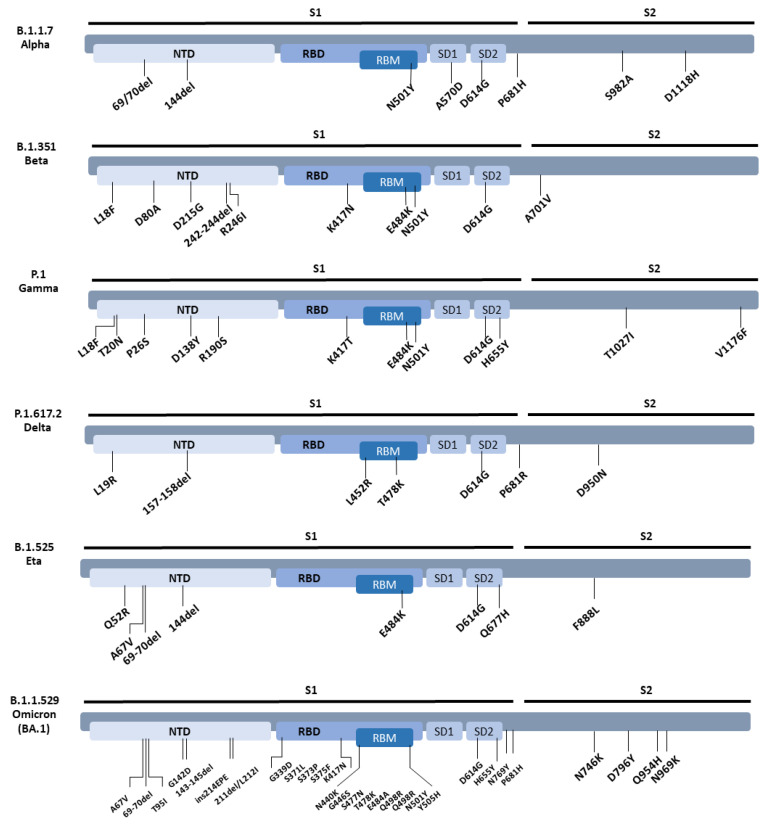
SARS-CoV-2 variants spike mutations used in this study.

At the start of the pandemic, there was a debate as to the possibility that antibodies raised against the HCOVs had any role in protection against SARS-CoV-2 [22,23,24,25]. Since then, rising interest in HCoVs has led to an increase in understanding of the immune response they generate. Several publications that investigated the effect of HCoVs relied on the use of binding assays such as enzyme linked immunosorbent assays (ELISA) that measure antibody binding but did not elucidate their neutralising capabilities. Moreover, successful generation of various vaccine platforms have been used to protect individuals from infection and severe disease [26], though their effectiveness is diminished as newer and more immune evasive variants arise [27]. Here, we use lentiviral-based pseudotyped viruses of SARS-CoV-2, the VOCs/VOI, and HCoVs, to measure the strength of neutralising antibodies induced by two doses of either BNT162b2 (Pfizer), AZD1222 (Astrazeneca), or mRNA-1273 (Moderna) against SARS-CoV-2 and variants B.1.1.7 (Alpha), B.1.351 (Beta), P.1 (Gamma), B.1.617.2 (Delta), B.1.525 (Eta), and B.1.1.529 (Omicron BA.1) (Figure 2), and whether any of these vaccines are able to augment neutralising antibodies against HCoVs 229E, HKU1, or NL63. 

## 2. Materials and Methods

### 2.1. Patient Serum Collection/Ethics Information

Sera samples were collected from 36 healthy vaccinated subjects. The study was approved by San Raffaele Scientific Hospital Ethical Committee (protocol number 68/INT/2020). All enrolled patients gave written, informed consent.

### 2.2. Phylogenetic Tree and Similarity Plot

A maximum likelihood phylogenetic reconstruction based on the spike gene codon alignment, was constructed using iqtree (version 1.6.12) [28] with 10,000 ultra-fast bootstrap replicates [29] and a TVM+F+I+G4 substitution model, selected using ModelFinder [30]. The sequence similarity plot was constructed by aligning spike protein sequences of SARS-CoV-2 (QHD43416.1), HKU1 (YP_173238.1) 229E (NP_073551.1) and NL63 (YP_003767.1), using mafft (version 7.453) [31] (genafpair option) and visualised using the D3 JavaScript package implemented in observable (https://observablehq.com/@spyros-lytras/seasonal-cov-spike accessed on 3 November 2022).

### 2.3. Tissue Culture

Human embryonic kidney 293T/17 (HEK293T/17) cells and human hepatocytes Huh-7 cells were maintained in DMEM supplemented with 10% foetal bovine serum and 1% penicillin/streptomycin. Chinese ovarian hamster (CHO) cells were maintained in Ham’s F12 supplemented with 10% foetal bovine serum and 1% penicillin/streptomycin. Cells were routinely passaged to prevent confluency by washing with phosphate-buffered saline solution and detached with trypsin-EDTA. All cells were incubated at 37 °C and 5% CO_2_.

### 2.4. Pseudotype Virus Production

All pseudotypes (PVs) were generated as previously described [32]. Briefly, 1000 ng of pc-DNA 3.1+ plasmid bearing the spike of ancestral SARS-CoV-2, variants Alpha, Beta, Delta, Gamma, Eta, Omicron Ba.1, or HCoVs 229E, HKU1, and NL63 was mixed with 1000 ng of p8.91 plasmid encoding the HIV Gag-pol and 1500 ng of pCSFLW plasmid containing the *Renilla firefly* luciferase reporter gene, and co-transfected onto HEK293T cells at 50% confluency in T-75 flasks using FuGENE-HD. HKU-1 required an additional step of adding 1.5 U of exogenous neuraminidase (Sigma) in 10 mL of replenished DMEM 24 h after transfection. To harvest the pseudotyped viruses, media was aspirated 48 h after day of transfection and filtered using a 0.45 µm cellulose acetate filter. All PVs were aliquoted and stored at −80 °C for storage. After repeated attempts, we were unable to pseudotype HCoV OC43.

### 2.5. Pseudotype Virus Titration

All PVs were titrated as previously described [32]. Target cells for SARS-CoV-2, variants, and HCoV NL63 were prepared the day before titration by transfecting ACE-2 and TRSSMP2. CHO cells were used as target cells for HKU-1, and Huh-7 cells were used as target cells for 229E. Briefly, 50 µL of harvested PV were added in the top row of a white F-bottom 96-well plate (Nunc), and serially diluted using DMEM or Ham’s F-12 for HKU-1 PVs in half steps to the bottom row of the plate prior to addition of 10,000 target cells in each well. Plates were returned to the incubator for 48 h prior to lysis with Bright-Glo reagent and assaying luciferase reporter gene activity in relative line units (RLU) using a Glo-Max luminometer. PV titres are reported in RLU/mL.

### 2.6. Pseudotype Microneutralisation (pMN) Assays

The pMN assay was carried out as previously described. Briefly, convalescent sera were mixed with either DMEM or Ham’s F-12 at an initial 1:40 dilution and then serially diluted 2-fold in a white flat-bottomed 96-well plate to a final dilution of 1:5120. All samples were repeated in duplicate. PVs were then added to each well at an input of 1 × 10^6^ RLU/mL. Plates were returned to the tissue culture incubator for 1 h, prior to addition of pre-transfected ACE-2/TRSSMP2 HEK293T target cells or CHO cells for HKU-1 and Huh-7 cells for 229E, at a density of 1 × 10^4^ cells per well. Plates were returned to the incubator for 48 h prior to lysis with Bright-Glo reagent and assaying luciferase reporter gene activity in relative line units (RLU) using a Glo-Max luminometer. IC50s were calculated using GraphPad Prism 8 software using a non-linear regression curve as described in [33].

### 2.7. Statistical Analysis

Wilcoxon matched-pair ranked tests were used to assess significance in matched subjects. Kruskal–Wallis ANOVA test was used to assess significance when comparing IC50 titres between three vaccine platforms. All tests were used on Graphpad Prism 8 software.

## 3. Results

### 3.1. Cohort Characteristics

To assess the neutralizing potential of SARS-CoV-2 specific antibodies against SARS-CoV-2 VOCs and hCoVs, sera obtained from double-dosed BNT162b2-vaccinated (n = 13), AZD1222-vaccinated (n = 16) and mRNA-1273-vaccinated (n = 7) individuals with and without an history of SARS-CoV-2 infection were inspected (Table 1).

### 3.2. Neutralisation of SARS-CoV-2 Variants

**Figure 3:**
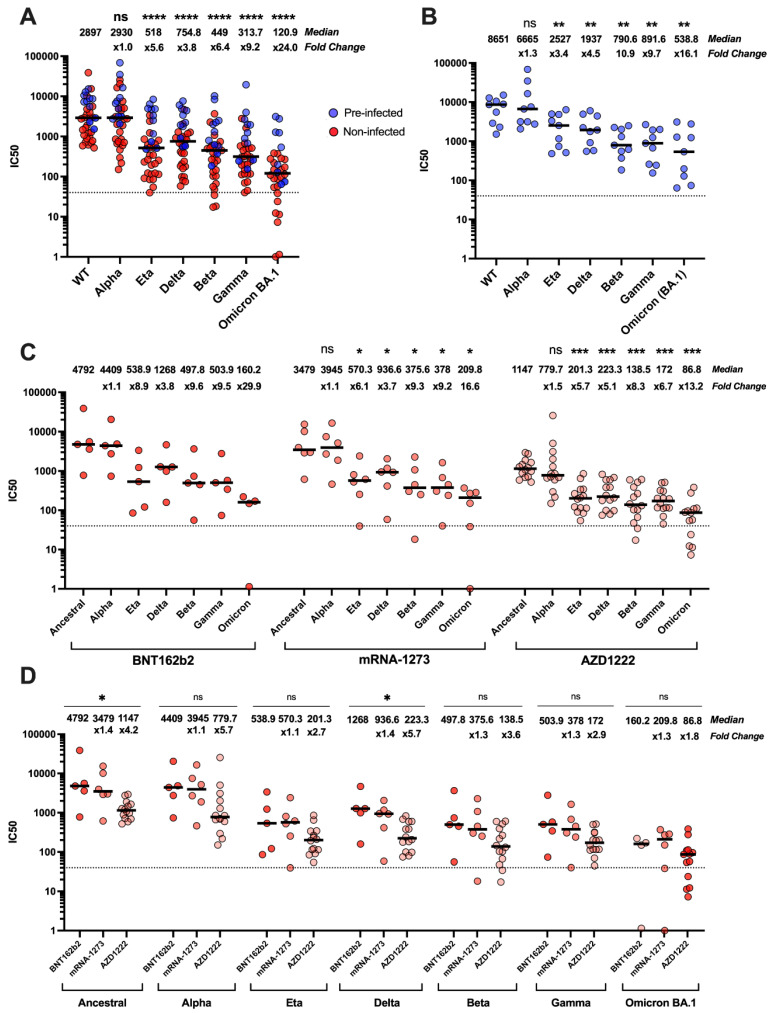
Ability of serum antibodies to neutralise SARS-CoV-2 and VOCs from individuals vaccinated with two doses of either BNT162b2, AZD1222, or mRNA-1273. Neutralizing antibody response against the ancestral SARS-CoV-2 and variants, in previously infected individuals (blue) and non-infected individuals (red) receiving two doses of either BNT162b2, AZD1222, or mRNA-1273 vaccines (**A**). Wilcoxon matched-pairs signed rank tests statistical analysis was used to compare ancestral SARS-CoV-2 against each variant (**A**). Neutralisation profiles of sera from BNT162b2-vaccinated subjects with a history of prior infection. No statistical test was used for BNT162b2 in panel C due to small sample size with large variation. (**B**). Neutralisation profiles of the three vaccine types against variants (**C**) and compared between vaccine platforms. (**D**) Wilcoxon matched-pairs signed rank tests statistical analysis was used to compare ancestral SARS-CoV-2 against each variant in panel C. Kruskal–Wallis ANOVA was used for statistical analysis in panel D. ns = not significant, * *p* < 0.05, ** *p* < 0.01, *** *p* < 0.001, **** *p* ≤ 0.0001.

We first carried out pMN assays to analyse the magnitude of neutralising antibody responses against ancestral SARS-CoV-2 and variants, irrespective of vaccine type (Figure 3A). Our results showed that Omicron BA.1 was the least neutralised VOC (24-fold decrease, *p* =< 0.0001). As expected, we observed the samples from individuals with prior infection had higher neutralisation titres compared with immunological naïve subjects.

The serum from previously infected individuals (Figure 3B), neutralized the Alpha variant more effectively compared with the ancestral strain, as it showed a 1.3-fold decrease in median IC_50_ titre, followed by Eta and Delta variants, (3.4- and 4.5-fold decrease, respectively). Beta and Gamma variants were more resistant to neutralization (10.9- and 9.7-fold decrease, respectively), and Omicron BA.1 reached a 16.1-fold decrease compared with ancestral SARS-CoV-2. Notably, the majority of these subjects had received the BNT162b2 vaccine.

Taken together, these results suggested that in vaccinated subjects the pre-existing immunity raised by natural infection with SARS-CoV-2, or a VOC is more effective in protecting against the spectrum of variants that emerged later over time, compared with immunity triggered by vaccination only. However, the recently emerged variants evolved mechanisms to evade the neutralizing antibody response.

We then analysed subjects who had not experienced SARS-CoV-2 infection before vaccine administration (Figure 3C). The efficacy of each vaccine platform was analysed with respect to the capability to neutralize both the ancestral strain and its variants. We observed that the sera from BNT162b2-vaccinated subjects had high median IC_50_ titres compared with those obtained from mRNA-1273- and AZD1222-vaccinated individuals. Whereas the Alpha variant did not show immune escape in any of the vaccinated subjects, all the VOCs were resistant to antibody neutralization to different degrees (Figure 3C). We were unable to extract meaningful significance scores from the BNT162b2 samples due to few samples (n = 5) with very large spread in IC_50_ titres.

We did not observe any statistically significant difference between the three vaccine platforms with respect to their abilities to neutralize the Alpha, Eta, Beta, Gamma, and BA.1 variants. Conversely, the biggest difference between the three vaccine types was observed with ancestral and Delta variant, as mRNA-1273 showed a 1.6- and 1.4-fold decrease in median IC_50_ titres, respectively, compared with BNT162b2, whereas AZD1222 showed a 3.8- and 4.1-fold decrease (Figure 3D).

### 3.3. Neutralisation of Seasonal HCoVs

**Figure 4:**
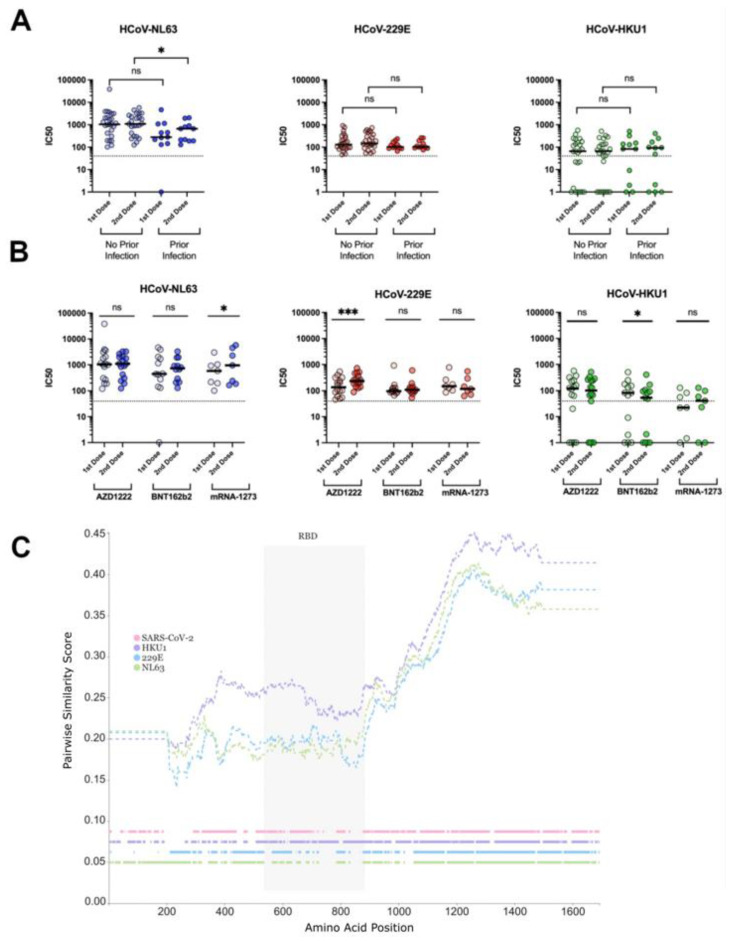
Comparing neutralising responses in HCoVs NL63, 229E, and HKU1 between first- and second-dose vaccination against SARS-CoV-2. Neutralization profile against HCoVs NL63, 229E, and HKU1 in double-dosed BNT162b2, mRNA-1273 or AZD1222 –vaccinated subjects with or without a history of SARS-CoV-2 infection. (**A**). Neutralizing antibody titres against the aforementioned HCoVs after the first and second dose administration of BNT162b2, mRNA-1273 or AZD1222 vaccines. (**B**). Wilcoxon matched-pairs signed rank tests statistical analysis was used in A and B. Similarity plots (**C**) show HKU-1 spike as having more similar amino acid sequence to SARS-CoV-2 compared with both NL63 and 229E in all regions of the spike protein. Dashed lines on the top show amino acid pairwise similarity between SARS-CoV-2 and the 3 HCoV Spike proteins, plotted using a 400 amino acid window size and a step of 1. Positions with gaps were excluded from the windows. Horizontal lines on the bottom indicate residue presence for each of the 4 aligned coronaviruses across the alignment length (colour presence = amino acid presence; colour absence = gap). ns = not significant, * *p* < 0.05, *** *p* < 0.001.

To determine whether vaccination against SARS-CoV-2 may cross-protect against seasonal HCoVs, we asked whether a prior infection with SARS-CoV-2 had any impact on antibody-mediated neutralisation of the HCoVs (Figure 4A). We observed no statistically significant increases in neutralizing titres against either 229E or HKU-1 between previously SARS-CoV-2 infected and naïve individuals. Conversely, a statistically significant decrease in neutralizing titres against NL63 after the second dose administration was found in vaccinated subjects who experienced SARS-CoV-2 infection (*p* = 0.033) compared with the naïve (*p* = 0.063).

We then assessed whether one or more of the vaccine platforms would boost titres against the HCoVs in all subjects, irrespective of their previous infection status (Figure 4B). Overall, in vaccinated subjects, the median antibody neutralization titres against NL63 were higher compared with those against 229E and HKU1, irrespective of the vaccine platform (Figure 4B). Notably, NL63 uses ACE2 as entry receptor into the target cells, as does SARS-CoV-2.

The type of vaccine did not have an impact in boosting the neutralizing activities against the three seasonal coronaviruses we studied after the second dose administration, with the exception of NL63 and HKU1. IC_50_ titres against NL63 increased after the second dose using mRNA-1273 (*p* = 0.03), whereas 229E showed a statistically significant increase in IC_50_ titre in only AZD1222-vaccinated individuals (*p* =< 0.001). Conversely, after the second boost of BNT162b2 vaccine, neutralization titres against HCoV HKU-1 decreased, probably due to the selection of antigen-specific plasma cells with lower affinity for the HKU1 spike.

To better understand the impact of COVID-19 vaccination on the protection from seasonal HCoVs in subjects with or without a history of SARS-CoV-2 infection, we analysed the spike protein similarity of HCoVs HKU1, NL63, 229E and SARS-CoV-2 to investigate whether a particular region could explain the neutralisation differences (Figure 4C). The similarity plot generated by comparing pairwise similarity showed HKU1 had higher similarity in all spike regions to SARS-CoV-2 spike compared with 229E and NL63, consistent with the viruses’ taxonomy. However, HKU1 seems to have extra insertions at the C-terminal end of the RBD compared with the other two seasonals and SARS-CoV-2. Furthermore, the S2 region shows much higher similarity to SARS-CoV-2 in all three HCoVs compared with the S1 region (Figure 4C).

## 4. Discussion

In this study, we were able to directly compare the antibody neutralisation titres induced by two m-RNA-based vaccines, BNT162b2 and mRNA-1273, and an adenoviral-based vaccine, AZD1222, against SARS-CoV-2, its emerged variants, and three seasonal HCoVs.

Our data on antibody neutralization against SARS-CoV-2 and its variants in vaccinated subjects, with or without a history of previous infection, agree with what is reported in the literature [2,4,5,7,34,35,36,37,38]. We confirmed that vaccination with two doses of vaccines induced antibodies able to neutralize SARS-CoV-2 and VOCs, with BNT162b2 eliciting the highest neutralization titres, followed by mRNA-1273 and AZD1222. Despite their differences in neutralization titres, all three vaccines have been reported to have high efficacy at preventing severe COVID-19 [39,40,41]. The Omicron BA.1 variant was the most evasive of all VOCs analysed in this study (Figure 3). Indeed, the heavily mutated spike protein of BA.1 variant posed challenges to the effectiveness of the current vaccines to protect against COVID-19 and pointed out the need to monitor the protection conferred against this and the newly emerged SARS-CoV-2 variants, namely Omicron BA.4 and BA.5. Bivalent formulations of mRNA-based vaccines, containing both the mRNA of the spike of the ancestral SARS-CoV-2 and the one in common between the BA.4 and BA.5 lineages have been designed and authorized in order to counteract the evasion of the immune response elicited by the original vaccine design.

HCoVs are globally distributed and believed to induce short-lasting protective antibodies [42]. Therefore, there is a high likelihood of reinfection remaining elevated, especially during the winter periods [13,43,44,45] despite high seroprevalence [43,45,46]. It is currently debated whether prior infection with seasonal HCoVs elicits cross-reactive antibodies against SARS-CoV-2, and more importantly, if this translates into protection against SARS-CoV-2. Cross-reactive antibodies [47,48,49,50,51,52,53,54] and T-cell responses [55,56,57,58,59,60,61] were detected in pre-pandemic sera and healthy donors; however, similar experimental approaches have shown the opposite to be true by other investigators [62]. In addition, in many of the aforementioned articles that revealed cross-reactive antibodies in pre-pandemic samples, the number of cross-reactive samples was a small portion of the total sera analysed, suggesting that cross-reactivity, whilst it exists, is low.

The same question has been raised about antibodies elicited by COVID-19 vaccines, with studies showing cross-reactive antibodies to some but not all the seasonal HCoVs [63,64,65]. SARS-CoV-2 spike protein vaccination was shown to induce cross-reactive antibodies to both *Alpha*- and *Betacoronaviruses* in macaques [66]. It is important to deduce whether cross-reactive antibodies translate into protective, neutralising antibodies against SARS-CoV-2. Some reports suggested that whilst there is a small boost in antibodies towards HCoVs during SARS-CoV-2 infection, they are not associated with protection [67]. Similarly, studies showed that prior infection with HCoVs did not protect against SARS-CoV-2 infection and disease [68,69].

We did not find any boost of neutralizing antibody titres against HKU1 in our cohort of SARS-CoV-2-vaccinated subjects, irrespective of their SARS-CoV-2 pre-infectious status, with the exception of subjects administered with BNT162b2. This is in contrast with two reports that observed a boost in HKU-1 titres post vaccination against SARS-CoV-2 by BNT162b2 [63,64]. Hicks et al. showed that antibodies reacting to HCoV-OC43 and HCoV-HKU1 had minimal cross-reactivity with SARS-CoV-2, in accordance with the sequence homology of these proteins [54]. Moreover, a previous SARS-CoV-2 infection did not boost the cross-neutralization against either HKU1 or the more phylogenetically related HCoV-229E (Figure 4A). One report suggested that HKU1 may have another candidate receptor that has yet to be identified, due to the presence of a putative RBD, distant from the sialic acid binding regions [70,71]. It should also be noted that neutralising ability might not only be dependent on the pairwise similarity between amino acids in the protein, but also short insertions and deletions that can alter the protein’s structural conformation. For instance, the HKU-1-specific insertion at the C-terminal end of the RBD (Figure 4C) might partly explain our neutralisation results.

Conversely, we found that the second dose administration in naïve subjects increased the protective antibody response against NL63 compared with that obtained in previously infected subjects receiving the same dose. This was probably due to the fact that additional exposures to the spike antigen did not have an effect on antibody neutralization against NL63.

The differences in antibody neutralization between the HCoVs may be due to the differences in the spikes used by the vaccination platforms. BNT162b27 encodes full-length spike with the K986P and V987P mutation sites to stabilize the pre-fusion conformation of the protein [72]. The mRNA-1273 vaccine contains the coding sequence for a spike glycoprotein stabilized by the same proline substitutions used in the BNT162b2 vaccine, with a transmembrane anchor and an intact S1-S2 cleavage site. The pre-fusion conformation is stabilized by the consecutive proline substitutions, which are located in the S2 subunit at the top of the central helix [73]. Conversely, a native-like spike is expressed by the AZD1222 vaccine. As our naïve subjects were administered with the AZD1222 vaccine, we can speculate that the native form of the spike protein triggered the development of higher neutralizing antibodies titres compared with that induced by the pre-fusion-stabilized protein.

Conversely, the second immunogenic exposure to SARS-CoV-2 spike boosted the neutralizing response against NL63 or 229E (Figure 4B), as has been previously reported [67], depending on the vaccine platform, irrespective of the pre-infection status. Interestingly, another report observed the same cross-neutralizing activity, though this was irrespective of vaccine platform [74]. We speculate that cross reactivity can arise due to similarity in epitopes in the receptor-binding motif (RBM) of NL63 to SARS-CoV-2, since both viruses share ACE-2 as their entry receptor [75]. Similarly, an epitope overlapping the S2 fusion peptide in 229E has been reported to elicit cross-reactivity against SARS-CoV-2 [48]. Song et al. described protective neutralizing antibodies targeting the S2 subdomain [53]. Furthermore, a report during the original SARS-CoV-1 outbreak also found cross reactive antibodies against NL63 and 229E [76], strengthening the hypothesis of shared epitopes between *Alphacoronaviruses* and *Betacoronaviruses*. The S protein of NL63 does not contain the furin-recognition site and is not cleaved during biogenesis [77]. Similarly, the spike protein expressed by the mRNA1273 vaccine lacks the cleavage site; therefore, the conformation of the protein might be similar and might trigger neutralizing antibodies against shared epitopes and that are boosted after a second exposure to the same antigen.

The antigenic nature of the spike protein expressed by the different vaccines, together with multiple conformations they can acquire, might affect the development of neutralizing antibodies with different affinities towards several epitopes in the spike protein. Since the AZD-1222 spike does not contain the two proline mutations to stabilise its spike into a trimeric pre fusion structure [78,79], the presence of a post fusion spike could potentially elicit a larger immune response towards epitopes in the S2 domain. This may explain why we did not observe any boost in neutralizing titres against 229E in either mRNA-based, pre-fusion-stabilized immunogen, vaccinated samples. Ultimately, despite observing a boost in titres, it is impossible for us to state whether this translates into protective titres since correlates of protection against SARS-CoV-2 have yet to be defined.

There are several limitations in our study to consider. Our data would have benefitted from larger numbers of samples in all vaccine platform types, and control samples of non-vaccinated individuals who either have been infected with SARS-CoV-2 or not. Furthermore, we did not analyse the baseline levels of cross-reactive neutralizing antibodies against seasonal coronaviruses in our cohort of vaccinated subjects.

A pan-coronavirus vaccine would elicit antibodies that recognise and neutralise a broad range of coronaviruses. This is challenging because of the genetic nature of these RNA viruses that frequently mutate and induce an immunity that wanes over time, increasing the likelihood of reinfection. Therefore, identifying the key epitopes located at the most conserved regions of the spike protein, especially at the S2 subunit, is relevant to potentially induce neutralizing antibodies with broader affinity to the cellular receptors that mediate viral entry. Several vaccine candidates have been formulated, and some are based on dual antigens including both spike and nucleocapsid (N) components [80]. These formulations are at the pre-clinical stage as they might provide broader and more durable humoral and cellular immune responses against coronaviruses [80].

## Figures and Tables

**Table 1:** Demographic and clinical characteristics of the cohort.

Total	36
Demographics	
Age (median [IQR], Range)	49 [43.5, 55.25] (24–62)
Sex (Male/Female)	8/28
SARS-CoV-2 Prior Infection (Yes/No)	11/28
BNT162b2 Samples (1st dose/2nd Dose)	13/13
mRNA-1273 Samples (1st dose/2nd Dose)	7/7
AZD1222 Samples (1st dose/2nd Dose)	16/16
Time of bleed after 1st dose	21 days (BNT162b2 and mRNA-1273) 12 weeks (AZD1222)
Time of bleed after 2nd dose	15 weeks

## Data Availability

All datasets are available by contacting the corresponding author upon kind request.
